# School Bag-Related Factors and Their Implications for Walking and Cycling to School among New Zealand Adolescents

**DOI:** 10.3390/ijerph182413125

**Published:** 2021-12-13

**Authors:** Sandra Mandic, Kaisa Kentala, Margaretha Liliana Situmorang, Mohammad Lutfur Rahman, Kimberley King, Enrique García Bengoechea, Ann-Maree Fox, Ricardo Oliveira, Kirsten J. Coppell

**Affiliations:** 1 School of Sport and Recreation, Faculty of Health and Environmental Sciences, Auckland University of Technology, Private Bag 92006, Auckland 1142, New Zealand; 2 AGILE Research Ltd., Wellington 6012, New Zealand; 3 Centre for Sustainability, University of Otago, P.O. Box 56, Dunedin 9054, New Zealand; kaisa.kentala@otago.ac.nz (K.K.); kim.king@otago.ac.nz (K.K.); annmareefox@gmail.com (A.-M.F.); 4 Department of Medicine, University of Otago, P.O. Box 56, Dunedin 9054, New Zealand; margaretha.situmorang@postgrad.otago.ac.nz (M.L.S.); kirsten.coppell@otago.ac.nz (K.J.C.); 5 School of Surveying, University of Otago, P.O. Box 56, Dunedin 9054, New Zealand; lutfur.rahman@postgrad.otago.ac.nz; 6 Physical Activity for Health Research Cluster, Health Research Institute, Department of Physical and Sport Sciences, University of Limerick, V94 T9PX Limerick, Ireland; enrique.garcia@ul.ie; 7 Research and Innovation Unit, Sport Ireland, D15 PN0N Dublin, Ireland; 8 Institute of Physical Education and Sports, University of Rio de Janeiro State, Rio de Janeiro 20550-013, Brazil; ricardobrandaorj@gmail.com

**Keywords:** adolescents, school, walking, cycling, settlement types, school bag

## Abstract

Excessive school bag weight may be a modifiable barrier to active transport to school. This study examined correlates of school bag weight and adolescents’ perceptions of excessive school bag weight for walking and cycling to school among New Zealand adolescents living in diverse settlement types. Adolescents (*n* = 1512; 15.0 ± 1.3 years) completed a questionnaire and had their bag weight (*n* = 1190) and body weight (*n* = 1038) measured. Adolescents using active transport and rural adolescents had lighter school bags compared to their counterparts. One-third of adolescents reported excessive school bag weight for walking (31.2%) and cycling (37.2%) to school. Positive correlates of relative school bag weight were female gender (regression coefficient (95% CI): 0.53 (0.13, 0.93)), and underweight (2.21 (1.39, 3.02)), whereas negative correlates were Māori ethnicity (−0.87 (−1.41, −0.32)), overweight (−1.84 (−2.35, −1.34)) and obesity (−3.57 (−4.26, −2.87)), and school location in small urban areas (−2.10 (−4.19, −0.01)), and rural settlements (−3.58 (−5.66, −1.49)). Older adolescents, females, those with greater relative school bag weight, and those experiencing school bag-related pain symptoms and/or fatigue were more likely to report excessive school bag weight for both walking and cycling to school. Future initiatives should target reducing excessive school bag weight, particularly in female and urban adolescents.

## 1. Introduction

The low levels of physical activity and sedentary lifestyles increasingly found among adolescents [1] globally contribute to poor health, including obesity and reduced psychosocial health [2]. Walking and/or cycling to school is a convenient way to integrate physical activity into adolescents’ daily lives and is associated with higher levels of physical activity and energy expenditure [3,4,5,6].

Actual and/or perceived excess school bag weight may be a modifiable barrier to active transport to school among adolescents [7]. The average school bag weight for adolescents varies between countries, ranging from 2.8 kg in Iran [8] to 6.6 kg in New Zealand [9]. In addition, evidence from developing countries suggests the school bags of children attending primary schools in city centre urban areas are heavier compared to those of their peers from schools in suburban and rural areas [10,11]. Whether adolescents’ school bag weights differ across settlement types is not currently known.

Previous studies have examined a range of issues related to school bags including the type of school bag [12,13], how a school bag is carried [12,13], school bag contents [14], the daily length of time a school bag is carried [13], access and usage of lockers at school [13], perceptions of school bag weight in general [12] as well as for walking and cycling to school [15], and the associations between the school bag weight and pain [10,13]. However, the impact of the recent requirements in some developed countries such as New Zealand for adolescents to bring their own electronic device to school on their bag weights and what adolescents carry to school remains unknown.

Most previous studies examining school bag weight have focused on the effects of school bag weight on pain [8,13,16]. While some cross-sectional studies have reported an association between school bag weight and musculoskeletal issues in children and adolescents [17], there are no universally agreed recommendations for school bag weight limits [18,19,20]. Recommended cut-off thresholds for school bag weight range from 5% to 20% of a child’s body weight [18,19,20], and some studies suggest different school bag weight limits for children of different ages [16] and a lower limit for girls than boys [8].

In addition, actual and perceived school bag weight may influence adolescents’ and their parents’ decisions as to whether adolescents walk and/or cycle to school. A New Zealand study conducted in an urban setting found that approximately two-thirds of parents perceived that adolescents’ school bags were too heavy to carry to school, and nearly two-thirds of adolescents perceived their school bags as too heavy to carry for walking or cycling to school [21]. Compared to active transport users, a greater proportion of adolescents and parents who used motorised transport to school perceived adolescents’ school bag weights as a barrier for walking or cycling to school, whereas the measured school bag weight did not differ between adolescents using active, motorised, or mixed modes of transport to school [21]. However, whether adolescents’ school bag weights differ by transport to school mode after accounting for age, gender, weight status, and settlement types remains unknown.

Therefore, the purpose of this study was to examine the school bag-related factors (including weight, contents, type, and method of carrying), correlates of school bag weight and the perceptions of school bag weight for walking and cycling to school among New Zealand adolescents attending schools in diverse settlement types.

## 2. Materials and Methods

### 2.1. Participants

Adolescents were recruited from 17 out of 27 secondary schools in the Otago region, New Zealand as part of the Built Environment and Active Transport to School (BEATS) Rural Study in 2018 (11 of 15 schools; Otago region, New Zealand) [5] and the BEATS Natural Experiment in 2020–2021 (6 of 12 schools; Dunedin, New Zealand) [22]. Detailed research methodology for both studies has been published elsewhere [5,22]. For both studies, adolescents (age 13 to 18 years; school years 9 to 13) were recruited through their school. Invitation letters with study information and consent forms were sent by each participating school to eligible students and their parents 2 to 4 weeks prior to the scheduled data collection date(s). Adolescents who signed consent participated in this study. Parental consent was not required. The study protocols for both studies were approved by the University of Otago Human Ethics Committee (BEATS Rural: 17/178; BEATS Natural Experiment: 17/188).

A total of 1575 adolescents had a valid consent and valid main study survey data (BEATS Rural: *n* = 959; BEATS Natural Experiment: *n* = 616). After excluding adolescents with missing (*n* = 21) and invalid (*n* = 7) questionnaire data about school bags and those boarding at their school (*n* = 35), data from 1512 adolescents were analysed.

### 2.2. Student Survey

Participating adolescents completed a 35–45 min online BEATS Student Survey [15] adapted for each study, as described previously [5,22]. This survey and a paper-based school bag questionnaire were completed during school time supervised by research staff. Survey items that related to sociodemographic characteristics (age, gender, ethnicity), adolescents’ travel to school behaviours [7], perceptions of walking and cycling to school [7], and self-reported physical activity [23] were used in this analysis. Home-to-school distance was determined using Geographic Information System (GIS) network analysis as the shortest path on a connected street network [15] in the BEATS Rural Study and using shortest walking distance from Google Maps in the BEATS Natural Experiment. Based on their school address, schools were categorised into one of the six urban and rural categories defined by Statistics New Zealand [24], then re-coded into four settlement types (‘large urban area’ (5 schools); ‘medium urban area’ (3 schools); ‘small urban area’ (4 schools), and ‘rural settlement’ (5 schools)) [25,26].

Adolescents self-reported frequency of use of different mode(s) of transport to school using response categories ‘never’, ‘rarely’, ‘sometimes’, ‘most of the time’, or ‘all of the time’ for each mode [7]. Based on the transport modes used ‘most of the time’, or ‘all of the time’, adolescents were categorised as users of ‘active transport’, ‘motorised transport’, or ‘mixed transport’ [7]. Using a 4-point Likert scale (1 = ‘strongly disagree’ to 4 = ‘strongly agree), adolescents also reported whether they perceived their home-to-school distance as being too far to walk or cycle to school [7]. Adolescents self-reported their physical activity using the question “Over the past 7 days, on how many days were you physically active for a total of at least 60 min per day?” [23,27].

### 2.3. School Bag Questionnaire

**Figure 1:**
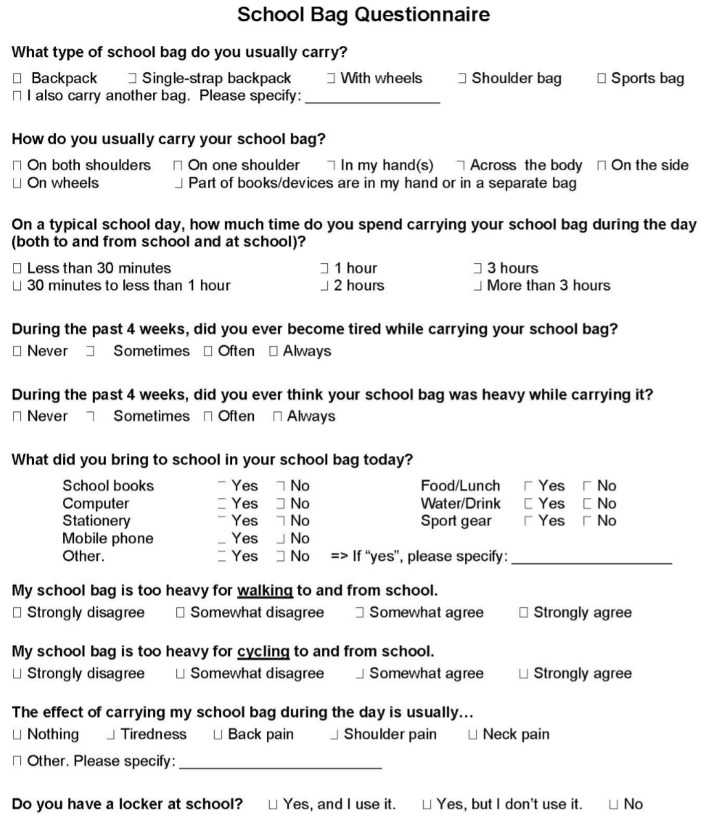
School bag questionnaire items. Notes: This figure does not contain images used in the study questionnaire as examples of different school bag types and different ways of carrying a school bag. In the BEATS Natural Experiment, the response categories to the question about a locker at school were ‘Yes, and I use it’, ‘Yes, but I don’t use it’, ‘No, but I want one’ and ‘No, and I don’t want one’ with the last two response categories recoded as ‘No’ for the analysis reported in this article.

Adolescents also completed a 1-page paper version of a school bag questionnaire designed specifically for the BEATS Rural Study and subsequently used in the BEATS Natural Experiment. The questionnaire was based on survey items adapted from previous studies (Figure 1) and included items related to school bag type [12], method of carrying the school bag [12,13], the amount of time spent carrying the school bag [13], being tired [12] or experiencing pain [8,28] from carrying a school bag, and accessibility to a locker at school [13]. Adolescents self-reported their school bag contents and their perceptions of their school bag weight in general [12] and specifically for walking and cycling to school [15]. The questionnaire was tested for comprehension by two adolescents and two parents of adolescents, then modified based on feedback prior to data collection.

### 2.4. School Bag Weight Measurements

At the time of the survey, research assistants weighed adolescents’ school bags using a digital electronic scale (A&D scale UC321, A&D Medical, San Jose, CA, USA), as described previously [21]. School bag measurements included the weight of each adolescents’ school bags without an electronic device and, if applicable, a separate weight measurement of their electronic device and/or any additional bags, including sports gear and/or musical instruments. Each weight was recorded to the nearest 0.01 kg. Adolescents self-reported whether their bag contained everything they had brought to school that day (‘full weight’) or not (‘partial weight’) [21].

### 2.5. Anthropometry

Adolescents’ height (custom-built portable stadiometer) and weight (A&D scale UC321, A&D Medical) were measured using standard measurement procedures, with adolescents wearing their school uniform (without a jacket, sweater, and shoes) [15,23]. Measurements were completed by trained research staff at the time of the survey in a screened off classroom area [15]. Height was measured to the nearest millimetre. Weight was recorded to the nearest 0.01 kg then reduced by 0.5 kg to account for clothing, as reported previously [23]. Both weight and height were measured twice, and the average was used in the analysis. International age- and sex-specific cut-off points for body mass index (BMI; weight divided by height squared (kg·m^−2^)) were used to determine adolescents’ body weight status categories, with BMI < 17 kg·m^−2^ indicating underweight [29].

### 2.6. Data Analysis

Sociodemographic characteristics were analyzed using descriptive statistics. Differences between groups were compared using Chi-square test for categorical variables and *t*-test or ANOVA for continuous variables with Scheffe post hoc multiple comparisons or Tamhane’s T2 test when the assumption of homogeneity of variance was violated. Correlates of adolescents’ relative school bag weights and their perceptions of excessive school bag weight for walking or cycling to school were examined using linear mixed models. Given a significant unconditional random intercept model to test whether relative school bag weight scores were correlated within schools (*p* < 0.001), subsequent models accounted for clustering effects (i.e., students nested within schools). The models assumed a variance components covariance type for the random intercept effects and used maximum likelihood as the estimating method. Variables with a significant effect in initial bivariate analyses (set at *p* < 0.10) were used to create multivariate models that provided the best fit to the data. In the multivariate models, a *p*-value < 0.05 was considered statistically significant. Data are reported as frequencies (percentage) for categorical variables and mean ± standard deviation (SD) for continuous variables. A *p*-value < 0.05 was considered statistically significant. Analysis was performed using SPSS software version 27.0.

## 3. Results

Data from 1512 adolescents (age: 15.0 ± 1.3 years; 51.4% female; 71.0% New Zealand European) were analysed (Table 1). One-third of adolescents attended school in a large urban area, 11.3% in medium urban areas, 43.9% in small urban areas, and 11.8% in rural settlements. Overall, 49.3% travelled to school using motorised transport, 26.2% used active transport, and 24.5% used mixed modes. 

Of the 1512 participants, 1421 (93.4%) had valid full or partial school bag weight data, 1190 (78.7%) had their full school bag weighed, and 1038 (68.7%) had relative school bag weight data (i.e., full school bag weight relative to adolescents’ body weights). The average school bag weight (including electronic devices but excluding other bags) was 5.2 ± 1.9 kg (Table 2), with no significant differences between genders (males: 5.2 ± 1.9 kg; females: 5.2 ± 1.9 kg; *p* = 0.481). The relative school bag weight was on average 8.8 ± 3.5% and significantly higher for female compared to male adolescents (females: 9.2 ± 3.8%; males: 8.4 ± 3.2%; *p* = < 0.001). In 98 adolescents who carried an additional bag to school, the average weight of the additional bag was 2.0 ± 1.5 kg (range: 0.4 to 9.9 kg). In 608 adolescents who carried their electronic device (e.g., laptop, Chromebook) to school, the average weight of devices was 1.6 ± 0.7 kg. Adolescents who carried an electronic device to school had a significantly heavier absolute (5.9 ± 1.8 vs. 4.5 ± 1.7 kg; *p* < 0.001) and relative school bag weight (9.8 ± 3.5% vs. 7.6% ± 3.2%; *p* < 0.001) compared to their counterparts. A greater proportion of adolescents aged 16 years and over carried an electronic device to school compared to their younger peers (58.8% vs. 49.1%, *p* < 0.010).

Most school bags were a backpack (92.1%), and 11.0% of adolescents reported bringing an extra bag to school. While most adolescents (85.3%) carried their school bag on both shoulders, 9.5% carried their school bag on one shoulder (females: 11.2%; males: 7.6%; *p* = 0.017). The contents of school bags included schoolbooks (86.4%), stationery (84.3%), food/lunch (87.3%), an electronic device (60.6%), water/drink (69.4%), a mobile phone (63.1%), and sports gear (55.6%).

**Figure 2:**
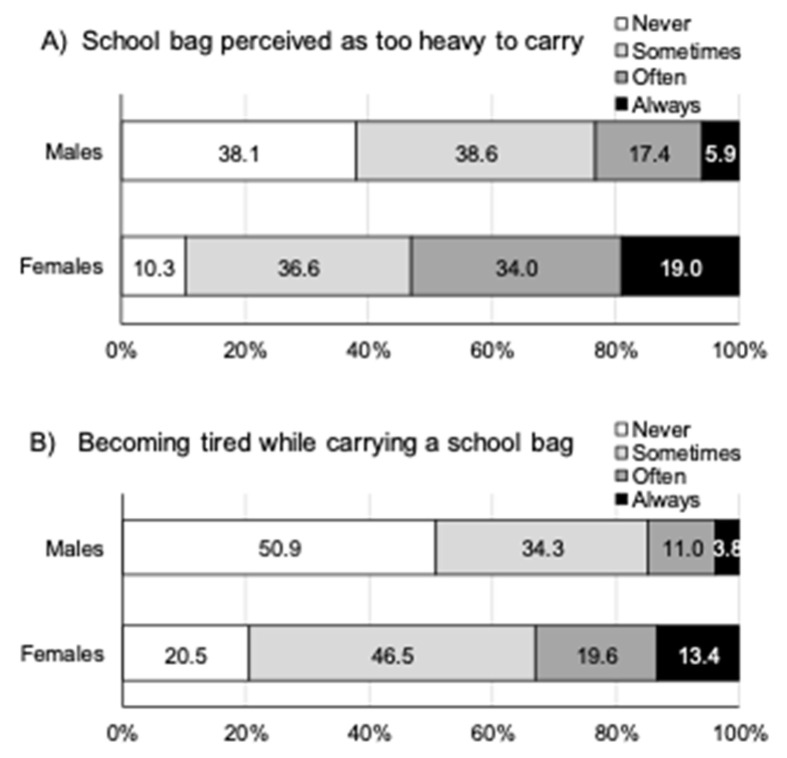
Adolescents’ perceptions of their school bag weight and effects on becoming tired.

On average adolescents reported carrying their school bags for 1.4 ± 1.2 h per day with no gender differences (*p* = 0.542). Overall, two-thirds reported becoming tired while carrying their school bag, and most adolescents thought their school bags were too heavy to carry (Table 2), especially females (Figure 2). Approximately one-third perceived their school bag to be too heavy for walking (31.2%) or cycling to school (37.2%), with twice as many females compared to males reporting this (for walking: 41.6% vs. 19.9%; for cycling: 48.7% vs. 23.7%; all *p* < 0.001).

Overall, 57.7% of adolescents reported pain and/or fatigue symptoms arising from carrying their school bag, with a significantly higher proportion of females compared to males (72.3% vs. 41.9%; *p* < 0.001). School bag-related fatigue and pain symptoms were more frequently reported by urban adolescents and those using motorised and mixed transport to school compared to rural adolescents and active transport users, respectively (Table 2). One-quarter of adolescents reported having and using a locker at school (24.1%), with a significantly greater proportion of female versus male adolescents (30.2% versus 17.2%; *p* < 0.001).

Active transport users had lower absolute and relative school bag weights than adolescents using motorised and mixed transport for school travel (Table 2). Active transport users also less frequently reported becoming tired while carrying their school bag, less frequently perceived their school bag to be too heavy for walking or cycling to school, and reported fewer pain symptoms and fatigue when carrying their school bag compared to their counterparts (Table 2).

On average, adolescents attending rural schools had the lowest absolute and relative school bag weights and carried their school bag for the shortest time compared to those in urban areas (Table 2). Overall, 36.4% of rural adolescents used a sport bag for their school bag compared with 5.6% to 11.1% of urban adolescents. A lower proportion of rural adolescents reported having school books, electronic devices, stationery, and mobile phones in their school bags compared to their urban peers.

### 3.1. Correlates of Relative School Bag Weight

Female adolescents and underweight adolescents had a higher likelihood of having heavier school bags relative to their body weight compared to males and healthy weight adolescents, respectively (Table 3). In contrast, Māori, overweight and obese adolescents, and those attending schools in rural settlements and small urban areas had a lower likelihood of carrying heavier school bags relative to their body weight compared to their counterparts (Table 3). Home-to-school distance and mode of transport to school were not significant univariate correlates of relative school bag weight and therefore were not included in the multivariate model.

### 3.2. Correlates of Perceived Excessive School Bag Weight for Walking to School 

For walking to school, older adolescents, females, adolescents with a greater relative school bag weight, and those self-reporting fatigue and/or pain symptoms from carrying their school bag were more likely to report excessive school bag weight for walking to school compared to their counterparts (Table 4). In contrast, adolescents who self-reported being more physically active were less likely to report excessive school bag weight for walking to school.

### 3.3. Correlates of Perceived Excessive School Bag Weight for Cycling to School

For cycling to school, older adolescents, females, adolescents with greater relative school bag weight, those reporting fatigue and/or pain symptoms from carrying their school bag, and those perceiving the distance to school as too far to cycle were more likely to report excessive school bag weight for cycling to school compared to their counterparts (Table 5).

## 4. Discussion

The key findings of this study are: (1) active transport users and rural adolescents had lighter school bags compared to their counterparts; (2) one-third of adolescents perceived their school bag to be too heavy for walking or cycling to school, and over half reported pain symptoms and/or fatigue arising from carrying their school bag; (3) female and underweight adolescents had a higher likelihood of having heavier school bags relative to their body weight, whereas Māori, overweight and obese adolescents, and those attending schools in small urban areas and rural settings had a lower likelihood compared to their counterparts; (4) older adolescents, females, those with greater relative school bag weight, and those who reported school bag-related fatigue and/or pain symptoms were more likely to report excessive school bag weight for both walking and cycling to school; (5) physically active adolescents were less likely to report excessive school bag weight for walking to school compared to their less physically active peers; and (6) adolescents who perceived the home-to-school distance as too far to cycle were also more likely to report excessive school bag weight for cycling to school. Taken together, these findings indicate that excessive school bag weight is a significant issue for adolescents, especially in some subgroups, and is a potentially modifiable barrier to active transport to school in adolescents. These findings have important implications for future strategies and interventions focused on encouraging walking and cycling to school among adolescents.

Most adolescents perceived their school bag to be too heavy to carry, and over half reported experiencing fatigue and/or pain symptoms due to carrying their school bag during the day. School bag-related fatigue and pain symptoms were more frequently reported by female adolescents, those attending schools in a large urban area, and by users of motorised and mixed transport to school compared to their counterparts. In the present study, 86.4% of adolescents had school books, and 60.6% had an electronic device in their school bags. Adolescents bringing an electronic device to school also had heavier bags and more frequently reported pain and/or fatigue symptoms as a result of carrying their school bag compared to their peers. Several previous studies have also found female adolescents more frequently reported fatigue and pain symptoms from carrying school bags than male adolescents [8,12,13,30]. However, not all previous cross-sectional studies observed an association between musculoskeletal complaints and school bag weight in children and adolescents [17]. A recent systematic review found no convincing evidence that school bag use was associated with an increased risk of back pain in this age group, but studies were either cross-sectional, and prospective studies were assessed as having a moderate or high risk of bias [31]. Although high-quality long-term follow-up studies are necessary [32], the results of this New Zealand study suggest that excessive school bag weight is an important health and wellbeing issue for New Zealand adolescents.

The findings of the present study also indicate that excessive school bag weight is a barrier that prevents New Zealand adolescents from walking and cycling to school. Adolescents who carried heavier school bags relative to their body weight and reported experiencing fatigue and/or pain symptoms arising from carrying their school bag were also more likely to perceive their school bag to be too heavy for both walking and cycling to school. However, physically active adolescents had a lower likelihood of perceiving their school bags to be too heavy for walking to school compared to their less physically active peers. Given the low levels of physical activity among adolescents globally [1], as well as in New Zealand [33], and the potential of active transport to school to increase these levels [3,4,5,6], programmes and strategies aimed at reducing adolescents’ school bag weights have the potential to facilitate active transport to school and increase physical activity in this age group, thereby improving adolescents’ health and wellbeing. 

This study suggests that excessive school bag weight is a significant issue for female adolescents in New Zealand. Compared to male adolescents, female adolescents had a higher likelihood of having heavier school bags relative to their body weight and reporting excessive school bag weight for both walking and cycling to school. In addition, a two to three times higher proportion of female adolescents reported fatigue and/or pain symptoms associated with carrying their school bags compared to their male counterparts, which is consistent with findings from previous studies [8,12,13,30]. However, two other studies reported lighter school bags among female versus male adolescents [12,21], and another found no gender difference in relative school bag weight [9]. Although there have been suggestions advanced for gender-specific school bag weight limits with a lower bag weight limit for girls versus boys [8], currently there is no universal agreement on the cut-off points for school bag weight limits for children and adolescents [18,19,20].

In this study underweight adolescents and those living in large and medium urban areas were more likely to have heavier school bags relative to their body weight compared to their healthy weight peers and those living in small urban areas and rural settings. In contrast, Māori adolescents were less likely to have heavier school bags compared to other ethnic groups. This could be at least in part explained by Māori adolescents having the lowest proportion of those who brought an electronic device to school compared to other ethnic groups (47.3% Māori vs. 51.6% to 61.5% in other ethnic groups), even though this difference was not statistically significant. Although age was not a significant multivariate correlate of relative school bag weight after accounting for other factors, older adolescents were more likely to report excessive school bag weight for walking and cycling to school. This could be partly explained by a higher proportion of older adolescents bringing an electronic device to school compared to their younger counterparts. Studies conducted in primary school children in Indonesia and Uganda found that school bag weights were higher among children attending schools in city centre urban areas compared to suburban and rural settings [10,11]. Several previous studies reported that younger adolescents and underweight adolescents had higher relative school bag weights than older adolescents and overweight and obese adolescents [8,9,21,34]. Overall, these findings suggest that future interventions aimed at reducing school bag weight in adolescents should target those living in urban areas, pay attention to underweight adolescents, and consider other relevant factors such as adolescents’ ages.

In the present study, adolescents using active transport to school on average had significantly lower absolute and relative school bag weight compared to their peers using motorised and mixed modes of transport. However, transport mode was not a significant correlate of relative school bag weight in a multivariate model. An earlier New Zealand study conducted in urban adolescents reported no difference in absolute and relative school bag weight by adolescents’ modes of transport to school [21]. Contrasting findings reported in this study could be in part explained by the inclusion of adolescents from different settlement types (including rural areas) and differences in absolute and relative school bag weight among settlement types. Therefore, future studies comparing school bag weight among adolescents travelling to school using different transport modes should also take into account settlement types as well as other relevant factors such as gender and individuals’ weight statuses.

To be effective, future programmes and strategies to promote walking and cycling to school should also take into account home-to-school distance [25], which is the strongest correlate of active transport to school among adolescents [35,36]. Although actual home-to-school distance was not related to adolescents’ school bag weights in the present study, adolescents who perceived themselves to be living too far away to cycle to their school were also more likely to report excessive school bag weight for cycling to school. Two recent studies reported differences in urban adolescents’ [37] and their parents’ [38] perceptions of walking and cycling to school based on how far they lived from school. Less than half of the parents living within walking distance to their adolescents’ schools in urban areas agreed that their adolescents had “too much stuff to carry to walk or cycle to school” [38]. The proportion of parents agreeing with that statement increased to over two-thirds among those living beyond walking but within cycling distance to school and over three-quarters of those living beyond cycling distance to school [38]. Therefore, future research examining adolescents’ perceptions of their school bag weight for walking or cycling to school should take into account both the objectively measured and perceived distance from home to school and consider other factors such as topography.

### 4.1. Implications

These findings have significant implications for future health promotion campaigns aimed at reducing adolescents’ school bag weights to encourage walking and cycling to school and address musculoskeletal pain. Reducing the amount of school books carriedto and from school is one example. Such interventions should consider age and focus on female and underweight adolescents and those living in large urban areas.

### 4.2. Study Strengths and Limitations

The study strengths of this research include a large representative sample of adolescents from 17 of 27 high schools located in diverse settlement types in one region of New Zealand, measured school bag weight and adolescents’ body weights, the use of a comprehensive school bag questionnaire designed specifically for this study, and school bag weight-related data analysis limited to participants who had the full weight of their school bag measured at the time of data collection. Study limitations include a cross-sectional study design which prevents making claims about causality, and potentially limiting the generalisability of study findings to other geographical locations in New Zealand and other countries. Future studies examining school bag weight as a barrier to walking and cycling to school among adolescents should also consider home-to-school distance, the topography of the route to school, and adolescents’ physical activity and fitness levels.

## 5. Conclusions

Excessive school bag weight for high school students is a significant issue and represents one of the barriers to active transport to school among New Zealand adolescents. Female and underweight adolescents and those living in large urban areas were more likely to have heavier school bags relative to their body weight compared to their counterparts. Older adolescents, females, those with greater relative school bag weight, and adolescents who experienced school bag-related fatigue and/or pain symptoms were more likely to report excessive school bag weight for walking and cycling to school. Future health promotion efforts should focus on designing strategies to reduce excessive school bag weight (both actual and perceived), particularly in female and urban adolescents.

## Figures and Tables

**Table 1:** Sociodemographic characteristics of study participants.

		Total Sample	Transport to School				Settlement Types				
			Motorised Transport	Active Transport	Mixed Modes	*p*-Value	Large Urban Area	Medium Urban Area	Small Urban Area	Rural Setting	*p*-Value
		n = 1512	n = 737	n = 391	n = 367		n = 509	n = 171	n = 656	n = 176	
Age (years)		15.0 ± 1.3	15.0 ± 1.4 ^c^	15.0 ± 1.3	14.8 ± 1.3 ^a^	0.015	14.7 ± 1.2 ^e,f,g^	15.1 ± 1.3 ^d^	15.1 ± 1.3 ^d^	15.3 ± 1.5 ^d^	<0.001
Gender (n(%))											
	Males	714 (47.2)	342 (46.4)	207 (52.9)	151 (41.1)		253 (49.7)	64 (37.4)	309 (47.1)	88 (50.0)	
	Females	777 (51.4)	382 (51.8)	180 (46.0)	212 (57.8)		237 (46.6)	106 (62.0)	346 (52.7)	88 (50.0)	
	Gender diverse	21 (1.4)	13 (1.8)	4 (1.0)	4 (1.1)	0.017	19 (3.7)	1 (0.6)	1 (0.2)	0 (0.0)	<0.001
Ethnicity (n(%))											
	New Zealand European	1070 (70.8)	543 (73.7)	258 (66.2)	260 (70.8)		327 (64.2)	119 (69.6)	488 (74.4)	136 (77.7)	
	Māori	216 (14.3)	86 (11.7)	62 (15.9)	64 (17.4)		85 (16.7)	23 (13.5)	83 (12.7)	25 (14.3)	
	Pacific	52 (3.4)	26 (3.5)	12 (3.1)	11 (3.0)		15 (2.9)	11 (6.4)	24 (3.7)	2 (1.1)	
	Asian	44 (2.9)	23 (3.1)	14 (3.6)	6 (1.6)		22 (4.3)	6 (3.5)	14 (2.1)	2 (1.1)	
	Other	129 (8.5)	59 (8.0)	44 (11.3)	26 (7.1)	0.036	60 (11.8)	12 (7.0)	47 (7.2)	10 (5.7)	0.001
Weight status (n(%))											
	Underweight	72 (5.5)	35 (5.5)	22 (6.3)	15 (4.6)		24 (5.9)	8 (4.8)	33 (5.6)	7 (4.5)	
	Healthy weight	929 (70.5)	447 (70.7)	249 (71.6)	223 (69.0)		281 (68.5)	115 (69.3)	420 (71.6)	113 (73.4)	
	Overweight	219 (16.6)	100 (15.8)	59 (17.0)	59 (18.3)		66 (16.1)	30 (18.1)	103 (17.5)	20 (13.0)	
	Obese	97 (7.4)	50 (7.9)	18 (5.2)	26 (8.0)	0.604	39 (9.5)	13 (7.8)	31 (5.3)	14 (9.1)	0.389
Number of vehicles at home (n(%))											
	None	26 (1.7)	10 (1.4)	6 (1.5)	10 (2.7)		15 (3.0)	2 (1.2)	7 (1.1)	2 (1.1)	
	One	279 (18.5)	117 (15.9)	89 (22.8)	70 (19.1)		131 (25.8)	22 (12.9)	101 (15.4)	25 (14.2)	
	Two or more	1205 (79.8)	610 (82.8)	296 (75.7)	287 (78.2)	0.025	361 (71.2)	147 (86.0)	548 (83.5)	149 (84.7)	<0.001
Settlement type for school location (n(%))											
	Large urban area	509 (33.7)	244 (33.1)	107 (27.4)	143 (39.0)		-	-	-	-	
	Medium urban area	171 (11.3)	94 (12.8)	34 (8.7)	41 (11.2)		-	-	-	-	
	Small urban area	656 (43.4)	304 (41.2)	201 (51.4)	151 (41.1)		-	-	-	-	
	Rural settlement	176 (11.6)	95 (12.9)	49 (12.5)	32 (8.7)	0.001	-	-	-	-	
Distance to school (km)		8.1 ± 10.6	11.5 ± 11.3 ^b,c^	1.3 ± 1.2 ^a,c^	8.4 ± 9.0 ^a,b^	<0.001	6.3 ± 6.8 ^f,g^	8.0 ± 10.4	8.3 ± 10.9 ^d^	10.3 ± 11.0 ^d^	<0.001
Usual transport to school habits (n(%))											
	Motorized transport	737 (49.3)	-	-	-		244 (49.4)	94 (55.6)	304 (46.3)	95 (54.0)	
	Active transport	391 (26.2)	-	-	-		107 (21.7)	34 (20.1)	201 (30.6)	49 (27.8)	
	Mixed modes	367 (24.5)	-	-	-		143 (28.9)	41 (24.3)	151 (23.0)	32 (18.2)	0.001

^a^ *p* < 0.05 vs. motorised transport; ^b^ *p* < 0.05 vs. active transport; ^c^ *p* < 0.05 vs. mixed transport; ^d^ *p* < 0.05 vs. large urban area; ^e^ *p* < 0.05 vs. medium urban area; ^f^ *p* < 0.05 vs. small urban area; ^g^ *p* < 0.05 vs. rural setting.

**Table 2:** School bag-related factors.

		Total Sample	Transport to School				Settlement Types				
			Motorised Transport	Active Transport	Mixed Modes	*p*-Value	Large Urban Area	Medium Urban Area	Small Urban Area	Rural Setting	*p*-Value
		n = 1512	n = 737	n = 391	n = 367		n = 509	n = 171	n = 656	n = 176	
School bag weight		(n = 1190)	(n = 585)	(n = 302)	(n = 286)		(n = 422)	(n = 126)	(n = 519)	(n = 121)	
	Absolute weight (kg)	5.2 ± 1.9	5.3 ± 1.9 ^b^	4.8 ± 1.8 ^a,c^	5.3 ± 2.0 ^b^	<0.001	5.4. ± 1.9 ^e,f,g^	6.0 ± 2.0 ^e,f,g^	5.0 ± 1.6 ^d,e,g^	4.2 ± 2.1 ^d,e,f^	<0.001
	Relative weight (% of body weight)	(n = 1038)	(n = 505)	(n = 270)	(n = 251)		(n = 343)	(n = 122)	(n = 466)	(n = 107)	
	Average	8.8 ± 3.5	8.9 ± 3.4 ^b^	8.2 ± 3.3 ^a,c^	9.2 ± 3.8 ^b^	0.004	9.4 ± 3.9 ^f,g^	9.9 ± 3.4 ^f,g^	8.5 ± 2.9 ^d,e,g^	6.7 ± 3.8 ^d,e,f^	<0.001
	<10.0% body weight (n(%))	709 (68.3)	338 (66.9)	194 (71.9)	167 (66.5)		217 (63.3)	68 (55.7)	332 (71.2)	92 (86.0)	
	10.0−14.9% body weight (n(%))	278 (26.8)	144 (28.5)	69 (25.6)	63 (25.1)		97 (28.3)	46 (37.7)	123 (26.4)	12 (11.2)	
	>15.0% body weight (n(%))	51 (4.9)	23 (4.6)	7 (2.6)	21 (8.4)	0.029	29 (8.5)	8 (6.6)	11 (2.4)	3 (2.8)	<0.001
Time spent carrying school bag during the day (h/day)		1.4 ± 1.2	1.3 ± 1.1 ^c^	1.4 ± 1.2	1.5 ± 1.2 ^a^	0.010	1.5 ± 1.2 ^g^	1.7 ± 1.3 ^g^	1.4 ± 1.2 ^g^	0.4 ± 0.5 ^d,e,f^	<0.001
During the past 4 weeks, did you ever become tired while carrying your school bag? (n(%))		(n = 1512)	(n = 737)	(n = 391)	(n = 367)		(n = 509)	(n = 171)	(n = 656)	(n = 176)	
	Never	521 (35.0)	254 (34.9)	155 (40.3)	106 (29.4)		89 (25.6)	33 (26.4)	182 (35.7)	75 (62.5)	
	Sometimes	604 (40.6)	292 (40.1)	169 (43.9)	137 (38.1)		139 (40.1)	59 (47.2)	217 (42.5)	33 (27.5)	
	Often	232(15.6)	120 (16.5)	36 (9.4)	73 (20.3)		72 (20.7)	24 (19.2)	72 (14.1)	7 (5.8)	
	Always	132 (8.9)	62 (8.5)	25 (6.5)	44 (12.2)	<0.001	47 (13.5)	9 (7.2)	39 (7.6)	5 (4.2)	<0.001
During the past 4 weeks, did you ever think your school bag was heavy while carrying it? (n(%))											
	Never	356 (23.7)	192 (22.1)	117 (30.2)	71 (19.5)		77 (22.2)	17 (13.6)	122 (23.6)	44 (36.7)	
	Sometimes	560 (37.3)	268 (36.6)	162 (41.9)	123 (33.7)		123 (35.4)	39 (31.2)	186 (36.0)	48 (40.0)	
	Often	393 (26.2)	202 (27.6)	78 (20.2)	110 (30.1)		92 (26.5)	51 (40.8)	140 (27.1)	20 (16.7)	
	Always	193 (12.8)	101 (13.8)	30 (7.8)	61 (16.7)	<0.001	55 (15.9)	18 (14.4)	69 (13.3)	8 (6.7)	<0.001
My school bag is too heavy for walking to and from school (n(%))											
	Strongly disagree	553 (36.8)	245 (33.5)	176 (45.2)	123 (33.6)		103 (29.4)	40 (32.0)	192 (37.1)	67 (56.3)	
	Somewhat disagree	481 (32.0)	232 (31.7)	135 (34.7)	109 (29.8)		117 (33.4)	38 (30.4)	159 (30.7)	33 (27.7)	
	Somewhat agree	373 (24.8)	105 (29.1)	66 (17.0)	110 (30.1)		99 (28.3)	41 (32.8)	139 (26.8)	15 (12.6)	
	Strongly agree	97 (6.4)	61 (8.3)	12 (3.1)	24 (6.6)	<0.001	31 (8.9)	6 (4.8)	28 (5.4)	3.44 ()	<0.001
My school bag is too heavy for cycling to and from school (n(%))											
	Strongly disagree	557 (37.3)	235 (32.4)	181 (46.6)	131 (36.0)		106 (30.6)	41 (33.3)	202 (39.1)	55 (47.0)	
	Somewhat disagree	386 (25.8)	188 (25.9)	108 (27.8)	84 (23.1)		83 (24.0)	33 (26.8)	140 (27.1)	30 (25.6)	
	Somewhat agree	376 (25.2)	201 (27.7)	74 (19.1)	100 (27.5)		104 (30.1)	31 (25.2)	124 (24.0)	24 (20.5)	
	Strongly agree	175 (11.7)	101 (13.9)	25 (6.4)	49 (13.5)	<0.001	53 (15.3)	18 (14.6)	51 (9.9)	8 (6.8)	0.010
The effect of carrying my school bag during the day is usually… (n(%))											
	No effect	630 (42.3)	297 (40.3)	203 (51.9)	128 (34.9)		175 (34.4)	60 (35.1)	290 (44.2)	114 (64.8)	
	Pain symptoms *	510 (34.2)	267 (36.2)	118 (30.2)	130 (35.4)		171 (33.6)	66 (38.6)	234 (35.7)	48 (27.3)	
	Fatigue	147 (9.9)	73 (9.9)	29 (7.4)	44 (12.0)		59 (11.6)	14 (8.2)	66 (10.1)	8 (4.5)	
	Pain symptoms * and fatigue	204 (13.7)	100 (13.6)	41 (10.5)	65 (17.7)	<0.001	104 (20.4)	31 (18.1)	66 (10.1)	6 (3.4)	<0.001

* Pain symptoms include shoulder, back and/or neck pain. ^a^ *p* < 0.05 vs. motorised transport; ^b^ *p* < 0.05 vs. active transport; ^c^ *p* < 0.05 vs. mixed transport. ^d^ *p* < 0.05 vs. large urban area; ^e^ *p* < 0.05 vs. medium urban area; ^f^ *p* < 0.05 vs. small urban area; ^g^ *p* < 0.05 vs. rural settings.

**Table 3:** Multivariate correlates of relative school bag weight in adolescents.

	Estimate	Std. Error	p-Level	95% Confidence Interval	
				Lower Bound	Upper Bound
Age	−0.02	0.07	0.756	−0.17	0.12
Gender (Ref. male)					
Female	0.53	0.20	0.009	0.13	0.93
Ethnicity (Ref. New Zealand European)					
Māori	−0.87	0.28	0.002	−1.41	−0.32
Pacific	−0.56	0.52	0.277	−1.57	0.45
Asian	0.72	0.54	0.183	−0.34	1.79
Other	0.28	0.34	0.412	−0.39	0.96
Weight status (Ref. healthy weight)					
Underweight	2.21	0.42	<0.001	1.39	3.02
Overweight	−1.84	0.26	<0.001	−2.35	−1.34
Obese	−3.57	0.36	<0.001	−4.26	−2.87
Settlement type (Ref. large urban area)					
Rural settlement	−3.58	0.98	0.002	−5.66	−1.49
Small urban area	−2.10	0.96	0.049	−4.19	−0.01
Medium urban area	−0.76	1.08	0.495	−3.07	1.56

**Table 4:** Multivariate correlates of adolescents’ perceptions of excessive school bag weight for walking and cycling to school.

	Estimate	Std. Error	*p*-Value	95% Confidence Interval	
				Lower Bound	Upper Bound
Age	0.06	0.02	0.001	0.02	0.10
Gender (Ref. male)					
Female	0.11	0.06	0.053	0.00	0.23
Relative school bag weight (% of body weight)	0.04	0.01	0.000	0.02	0.05
Number of days being physically active for 60 min per day	−0.03	0.01	0.043	−0.05	0.00
Being tired from carrying school bag (Ref. never)					
Always	1.01	0.12	0.000	0.77	1.24
Often	0.81	0.10	0.000	0.61	1.00
Sometimes	0.38	0.07	0.000	0.23	0.52
Effects of carrying school bag (Ref. none)					
Pain symptoms and/or fatigue	0.51	0.07	0.000	0.37	0.65

**Table 5:** Multivariate correlates of adolescents’ perceptions that their school bag is too heavy for cycling to school.

	Estimate	Std. Error	*p*-Value	95% Confidence Interval	
				Lower Bound	Upper Bound
Age	0.09	0.03	0.002	0.03	0.14
Gender (Ref. male)					
Female	0.22	0.08	0.006	0.06	0.38
Relative school bag weight (% of body weight)	0.05	0.01	0.000	0.03	0.07
Number of days being physically active for 60 min per day	−0.02	0.02	0.173	−0.06	0.01
It is too far to cycle to school	0.12	0.04	0.002	0.04	0.19
Being tired from carrying school bag (Ref. never)					
Always	0.70	0.16	0.000	0.39	1.02
Often	0.58	0.14	0.000	0.31	0.84
Sometimes	0.16	0.11	0.127	−0.05	0.37
Effects of carrying school bag (Ref. none)					
Pain symptoms and/or fatigue	0.51	0.10	0.000	0.31	0.71
Transport to school (Ref. motorised)					
Active transport	0.05	0.10	0.602	−0.14	0.24
Mixed transport	0.06	0.10	0.567	−0.13	0.25

## Data Availability

Data used in data analysis for this project will not be shared due to the sensitivity of the collected data as well as participants having been given assurances that the collected data will not be shared. Due to sensitivity of the collected data, BEATS research data will not be shared.

## References

[1] Guthold R., Stevens G.A., Riley L.M., Bull F.C. (2019). Global trends in insufficient physical activity among adolescents: A pooled analysis of 298 population-based surveys with 1.6 million participants. Lancet Child. Adolesc. Health.

[2] Tremblay M.S., LeBlanc A.G., Kho M.E., Saunders T.J., Larouche R., Colley R.C., Goldfield G., Gorber S.C. (2011). Systematic review of sedentary behaviour and health indicators in school-aged children and youth. Int. J. Behav. Nutr. Phys. Act..

[3] Faulkner G.E.J., Buliung R.N., Flora P.K., Fusco C. (2009). Active school transport, physical activity levels and body weight of children and youth: A systematic review. Prev. Med..

[4] Kek C.C., Bengoechea E.G., Spence J.C., Mandic S. (2019). The relationship between transport-to-school habits and physical activity in a sample of New Zealand adolescents. J. Sport Health Sci..

[5] White B., Bengoechea E.G., Spence J.C., Coppell K., Mandic S. (2021). Comparison of physical activity patterns across large, medium and small urban areas and rural settings in the Otago region, New Zealand. N. Z. Med. J..

[6] Khan A., Mandic S., Uddin R. (2020). Association of active school commuting with physical activity and sedentary behaviour among adolescents: A global perspective from 80 countries. J. Sci. Med. Sport.

[7] Mandic S., Hopkins D., Bengoechea E.G., Flaherty C., Williams J., Sloane L., Moore A., Spence J.C. (2017). Adolescents’ perceptions of cycling versus walking to school: Understanding the New Zealand context. J. Transp. Health.

[8] Dianat I., Sorkhi N., Pourhossein A., Alipour A., Asghari-Jafarabadi M. (2014). Neck, shoulder and low back pain in secondary schoolchildren in relation to schoolbag carriage: Should the recommended weight limits be gender-specific?. Appl. Ergon..

[9] Whittfield J.K., Legg S.J., Hedderley D.I. (2001). The weight and use of schoolbags in New Zealand secondary schools. Ergonomics.

[10] Mwaka E.S., Munabi I.G., Buwembo W., Kukkiriza J., Ochieng J. (2014). Musculoskeletal pain and school bag use: A cross-sectional study among Ugandan pupils. BMC Res. Notes.

[11] Layuk S., Martiana T., Bongakaraeng B. (2020). School bag weight and the occurrence of back pain among elementary school children. J. Public Health Res..

[12] Kellis E., Emmanouilidou M. (2010). The Effects of Age and Gender on the Weight and Use of Schoolbags. Pediatr. Phys. Ther..

[13] Grimmer K., Williams M. (2000). Gender-age environmental associates of adolescent low back pain. Appl. Ergon..

[14] Giusti P.H., de Almeida H.L., Tomasi E. (2008). Weight excess of school materials and its risks factors in south Brazil. A cross sectional study. Eur. J. Phys. Rehabil. Med..

[15] Mandic S., Williams J., Moore A., Hopkins D., Flaherty C., Wilson G., Bengoechea E.G., Spence J.C. (2016). Built Environment and Active Transport to School (BEATS) Study: Protocol for a cross-sectional study. BMJ Open.

[16] Adeyemi A.J., Rohani J.M., Rani M.A. (2014). Back pain arising from schoolbag usage among primary schoolchildren. Int. J. Ind. Ergon..

[17] Trevelyan F.C., Legg S.J. (2011). Risk factors associated with back pain in New Zealand school children. Ergonomics.

[18] The American Academic of Pediatrics (2017). Back to School Tips from the American Academy of Pediatrics. https://www.aap.org/en-us/about-the-aap/aap-press-room/news-features-and-safety-tips/pages/back-to-school-tips.aspx.

[19] Rateau M.R. (2004). Use of backpacks in children and adolescents. A potential contributor of back pain. Orthop. Nurs..

[20] Moore M.J., White G.L., Moore D.L. (2007). Association of Relative Backpack Weight with Reported Pain, Pain Sites, Medical Utilization, and Lost School Time in Children and Adolescents. J. Sch. Health.

[21] Mandic S., Keller R., Bengoechea E.G., Moore A., Coppell K.J. (2018). School bag weight as a barrier to active transport to school among New Zealand adolescents. Children.

[22] Mandic S., Hopkins D., Bengoechea E.G., Moore A., Sandretto S., Coppell K., Ergler C., Keall M., Rolleston A., Kidd G. (2020). Built environment changes and active transport to school among adolescents: BEATS Natural Experiment study protocol. BMJ Open.

[23] Mandic S., Bengoechea E.G., Coppell K.J., Spence J.C. (2017). Clustering of (un)healthy behaviors in adolescents from Dunedin, New Zealand. Am. J. Health Behav..

[24] Stats N.Z. (2018). Anzlic Metadata Urban Rural. https://datafinder.stats.govt.nz/document/21473-anzlic-metadata-2018-urban-rural/.

[25] Mandic S., Bengoechea E.G., Hopkins D., Coppell K., Smith M., Moore A., Keall M., Ergler C., Sandretto S., Wilson G. (2021). Examining the transport to school patterns of New Zealand adolescents by home-to-school distance and settlement types.

[26] Rahman M.L., Pocock T., Moore A., Mandic S. (2020). Active Transport to School and School Neighbourhood Built Environment across Urbanisation Settings in Otago, New Zealand. Int. J. Environ. Res. Public Health.

[27] Currie C., Gabhainn S.N., Godeau E., The International HBSC Network Coordinating Committee (2009). The Health Behaviour in School-Aged Children: WHO collaborative cross-national (HBSC) study: Origins, concept, history and development 1982–2008. Int. J. Public Health.

[28] Shamsoddini A., Hollisaz M., Hafezi R. (2010). Backpack Weight and Musculoskeletal Symptoms in Secondary School Students, Tehran, Iran. Iran. J. Public Health.

[29] Cole T.J., Bellizzi M.C., Flegal K., Dietz W.H. (2000). Establishing a standard definition for child overweight and obesity worldwide: International survey. BMJ.

[30] Navuluri N., Navuluri R.B. (2006). Study on the relationship between backpack use and back and neck pain among adolescents. Nurs. Health Sci..

[31] Yamato T.P., Maher C.G., Traeger A.C., Wiliams C.M., Kamper S.J. (2018). Do schoolbags cause back pain in children and adolescents? A systematic review. Br. J. Sports Med..

[32] Dockrell S., Simms C., Blake C. (2013). Schoolbag Weight Limit: Can It Be Defined?. J. Sch. Health.

[33] Smith M., Ikeda E., Hinckson E., Duncan S., Maddison R., Meredith-Jones K., Walker C., Mandic S. (2018). New Zealand’s 2018 Report Card on Physical Activity for Children and Youth.

[34] Aprile I., Di Stasio E., Vincenzi M.T., Arezzo M.F., De Santis F., Mosca R., Briani C., Di Sipio E., Germanotta M., Padua L. (2016). The relationship between back pain and schoolbag use: A cross-sectional study of 5318 Italian students. Spine J..

[35] Ikeda E., Stewart T., Garrett N., Egli V., Mandic S., Hosking J., Witten K., Hawley G., Tautolo E.-S., Rodda J. (2018). Built environment associates of active school travel in New Zealand children and youth: A systematic meta-analysis using individual participant data. J. Transp. Health.

[36] Mandic S., de la Barra S.L., Bengoechea E.G., Stevens E., Flaherty C., Moore A., Middlemiss M., Williams J., Skidmore P. (2015). Personal, social and environmental correlates of active transport to school among adolescents in Otago, New Zealand. J. Sci. Med. Sport.

[37] Mandic S., Bengoechea E.G., Hopkins D., Coppell K., Spence J.C. (2022). Adolescents’ perceptions of walking and cycling to school differ based on how far they live from school. J. Transp. Health.

[38] Mandic S., Hopkins D., Bengoechea E.G., Flaherty C., Coppell K., Moore A., Williams J., Spence J.C. (2020). Differences in parental perceptions of walking and cycling to high school according to distance. Transp. Res. Part F Traffic. Psychol. Behav..

